# Physical Layer Components Security Risks in Optical Fiber Infrastructures

**DOI:** 10.3390/s22020588

**Published:** 2022-01-13

**Authors:** Vladimir Spurny, Petr Munster, Adrian Tomasov, Tomas Horvath, Edvin Skaljo

**Affiliations:** 1 Department of Telecommunication, Brno University of Technology, Technicka 12, 616 00 Brno, Czech Republic; xspurn07@vut.cz (V.S.); tomasov@vut.cz (A.T.); horvath@vut.cz (T.H.); 2 Department of Physics, University of Sarajevo, 71000 Sarajevo, Bosnia and Herzegovina; skaljo@hotmail.com

**Keywords:** leakage, eavesdropping, crosstalk, data, splitter, multiplexer, optical network

## Abstract

Optical fiber communications are essential for all types of long- and short-distance transmissions. The aim of this paper is to analyze the previously presented security risks and, based on measurements, provide the risk level evaluation. The major risk is the possibility of inserting a splitter into the optical distribution network and capturing a portion of the entire spectrum, i.e., all channels in the optical fiber. Another significant security risk is crosstalk on multiplexers in networks with wavelength division multiplexing. The paper covers the macrobend attenuation evaluation of fiber and back-reflection measurements. Based on the measurements, risks were evaluated for both point-to-point and point-to-multipoint networks and, lastly, the paper covers crosstalk measurements of an optomechanical switch. Finally, all individual risks are evaluated according to the severity, and a proposal for risk minimization is provided.

## 1. Introduction

Optical networks are considered secure, and data transmission is protected from eavesdropping due to the principle of signal transmission in optical fibers. However, there are several ways to obtain part of a signal from a fiber without the user finding out that it is being tapped [1]. Although eavesdropping on a physical layer seems impossible and complicated, the fact that similar things happen in practice is evident, e.g., from [2], and it includes both singlemode and multimode fibers [3]. Basic methods are generally known [4], but no measurement has yet been made to confirm or refute the possibility of information leakage without disclosure. Although data are now encrypted [5], due to quantum computers, it is necessary to take into account that current encryption methods will be insufficient in the future. Physical layer eavesdropping, data leakage, and data security will need to be protected through quantum key distribution [6].

As authors Zafar M. Iqbal, H. Fathallah, and Nazih Belhadj in [7] state, there exist several types of fiber tapping methods. The paper focuses on verifying the possibility of data leakage using macro bending but does not cover the influence of the fiber bend radius on the attenuation. Moreover, the splitter insertion method is described as being possible but the practical measurement is lacking. These methods with different approaches are measured by M. Diaa, M. Shalaby, A. A. Mohamed, A. Mokhtar, and K. Hassan in [8]. In the paper [9] from Akpan Aniefioks, the author theoretically describes common methods of fiber tapping but without practical verification. Interchannel eavesdropping in wavelength-division multiplexing (WDM) systems is theoretically described in S. Yuan and D. Stewart’s paper [10] but a practical comparison of crosstalk for different types of devices used in WDM networks with the thorough determination of the effect of source power is missing. At the same time, there is no practical proof of crosstalk in WDM ADD/DROP MUX components.

The purpose of this paper is to present the widest preview of optical fiber vulnerabilities and to examine the possibility of carrying it out in practice. The paper also provides risk analysis for every measured method and gives comprehensive risk minimization options.

A risk assessment system is used for risk analysis. In total, it consists of four categories, and each category can reach values from 1 to 5. Subsequently, the points from each category are added together. Thus, each risk can be assessed using the resulting risk value, which in the worst case can be 20. Conversely, a value of 0 means zero risk. The first category evaluates the invasiveness of the attack, i.e., whether there will be a route interruption or increase in attenuation. The second category evaluates the type of information leak, and whether all data are encrypted, some of the data are encrypted, or encryption is not used at all. The next category gives information about the complexity of the attack and the degree of knowledge of the attacker necessary to carry out the attack. The last category deals with the complexity of attack detection. Attacks of 13 or more should be given special attention as they pose a very high risk.

In Section 2, we describe data leakage methods with and without line/route interruption. Section 3, Section 4, Section 5, Section 6 and Section 7 are focused on practical measurement of selected methods with risk analysis and a risk minimization subsection at the end of each section. Basic schemes are also included. Section 8 concludes the paper.

## 2. Data Leakage in Optical Networks

There are many possible ways in which information can leak from the optical fiber infrastructure. It is not easy to classify leakage methods but the basic division can be made as follows. The first group includes attacks in which link/route interruption occurs; thus, there are noticeable mechanisms to control the network. Another group is based on attacks that do not require a change in the optical fiber infrastructure, especially the various interception options due to the physical properties of optical fibers [11,12,13,14,15,16].

Possibilities of data leakage with line interruption include methods such as:

- inserting a splitter/coupler;
- inserting a demultiplexing component;
- inserting an active device (a technique replicates the classic man-in-the-middle attack and is based on inserting an active device between two other devices communicating with each other).

Possibilities of data leakage without line interruption include methods such as:

- fiber tapping;
- back-reflection measurement;
- crosstalk between channels;
- malicious devices (in passive optical networks);
- monitoring ports of active devices.

## 3. Inserting an Optical Splitter/Coupler

**Figure 1:**
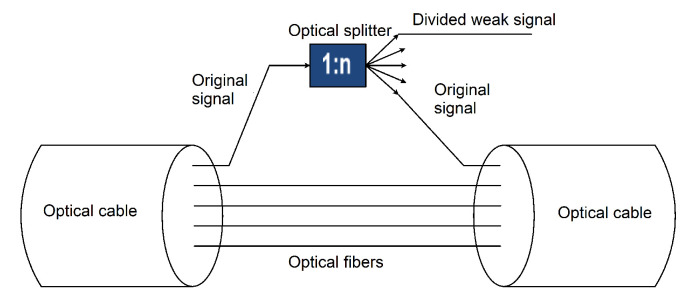
Data leakage by inserting a splitter into the ODN.

An optical splitter is a passive component that divides the input signal into *n* output signals (see Figure 1). For insertion, the route must be disconnected, and a splitter (or coupler) is inserted into the optical path. Depending on its duration, outage can be very easily detectable by a network monitoring system on the provider’s side. The malfunction of services on the side of the user can be negligible unless it is a passive optical network (PON), where the entire network must be rebuilt, which means a long outage. This can be bypassed by the correct timing of the attack—for example, in parallel with a planned or accidental outage, when someone else breaks the route. An inserted splitter/coupler can have an asymmetric division ratio, up to 99:1, which means that the insertion loss into the optical path is very small, and, when installed correctly, the splitter is almost undetectable.

In case of 99:1 division ratio splitters/couplers, the attenuation can be so small that even when measuring the insertion loss by the insertion loss (IL) measurement method (also called reference method), detection may not occur. For optical time-domain reflectometry (OTDR) measurements, detection may occur if the splitter is not located appropriately in the optical distribution network (ODN), i.e., at the beginning/end of the route, near another splitter, etc. Eavesdropping through optical splitters/couplers is very effective, and these splitters can serve for several years without any detection. This type of attack can have many variations depending on the location of the splitter in the ODN. It is possible to insert the splitter behind other elements, causing attenuation or amplifying the signal and thus further reducing the chance for detection.

### 3.1. Insertion Loss Measurement in ODN

The aim of the measurement was to determine the insertion loss of inserted splitters/couplers with ratios of 80:20, 90:10, and 99:1, respectively. Couplers with FC/APC connectors were used. Between each connection, the connectors were cleaned and inspected with a microscope. Dirty connectors can cause attenuation of approximately 0.3 dB, so cleaning is essential. Measurement at wavelengths of 1310 and 1550 nm, with subsequent analysis of information leakage, including real implementation of the attack with time estimation needed for the attack, is presented here. The results of measurements for the insertion loss method for all couplers are shown in Table 1, Table 2 and Table 3. Results for the optical time-domain reflectometry (OTDR) method are given in Table 4, Table 5 and Table 6.

An EXFO FLS-600 light source and an FOPM-102 optical power meter were used for insertion loss measurement.

**Figure 2:**
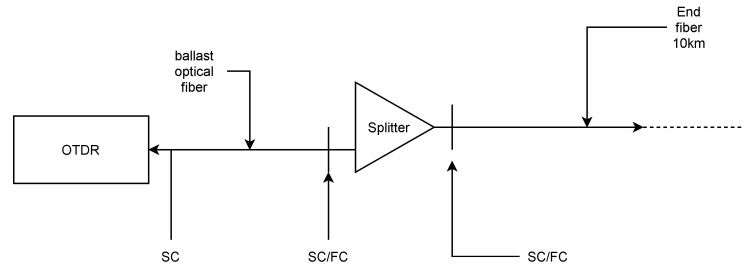
OTDR measurement setup.

OTDR analyzes and evaluates the parameters of the Rayleigh scattering, which reflects back a part of the light emission. It also analyzes the parameters of the Fresnel reflections created by the boundaries of two distinct optical environments with different refractive indexes. The light rays are attenuated by the rising length of the fiber. Optical routes are analyzed by OTDR, creating a curve showing all irregularities in the fiber path and their positions (fiber splice, splitters, etc.) [3]. For the experiment, we used an OTDR Atomo wave SOT-A80, 500 m of launch fiber with SC/APC connectors, splitters, SC/FC adapters, and 10 km of reference fiber spool (see Figure 2). The connectors were cleaned during all experiments.

### 3.2. Attack Time Duration Measurement

This section evaluates the time duration required to perform the attack. The attack scenario consists of splitter insertion into specific network segments, where it is possible to disconnect it using existing connectors (e.g., a network segment with a connection point or an amplifier). Next, a similar scenario is a splitter insertion close to another existing splitter in the network. This scenario simulates a typical setup in rural areas, in which the fiber infrastructure is located in a house cellar together with other infrastructure.

#### 3.2.1. Point-to-Point Network Topology

**Figure 3:**
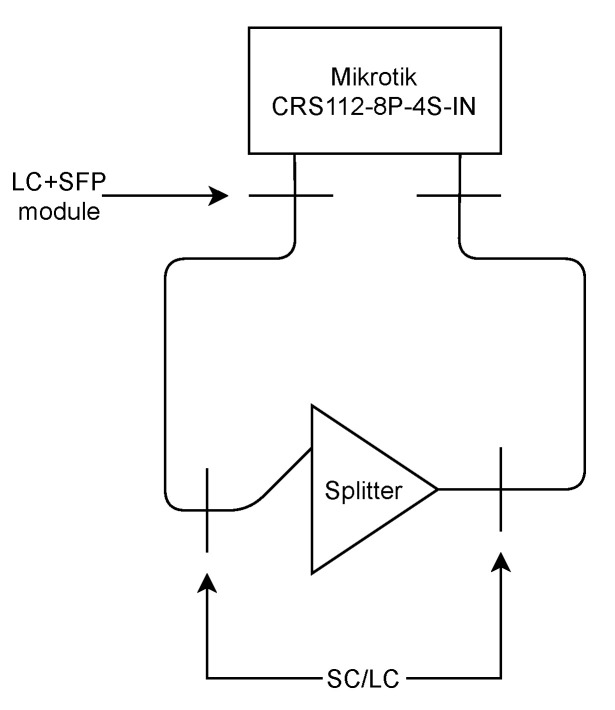
P2P—time of attack measurement setup.

As part of the measurement, it was necessary to ensure accurate measurement of time windows for outages. The Mikrotik CRS112-8P-4S-IN, which has 4 small form-factor pluggable (SFP) ports, was used for this measurement (see Figure 3). Thanks to the complexity of the used system, it was possible to measure precisely the outage duration, directly on the physical layer. A total of 30 measurements were performed, followed by calculation of the arithmetic mean and a median. According to the results, the time required to carry out the attack was determined to be 2.9 s in the case of the arithmetic mean and 3.0 s as the median value. Measurements were carried out under ideal conditions, with individual components placed on a table for the fastest possible switching.

#### 3.2.2. Point-to-Multipoint Network Topology

**Figure 4:**
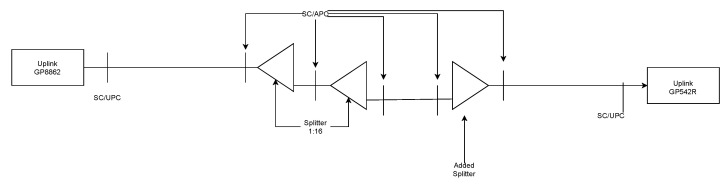
GPON—time of attack measurement setup.

For the comparison, another time measurement was performed on the existing gigabit passive optical network (GPON) infrastructure (the measurement scheme is shown in Figure 4).

**Figure 5:**
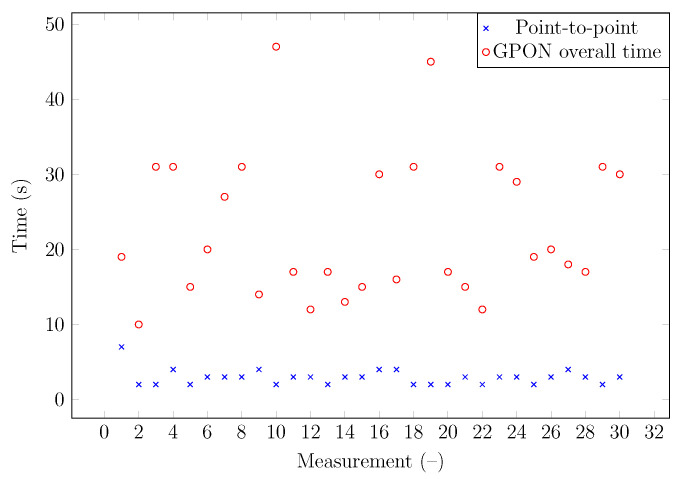
Time for reactivation of observed ONU.

Two values of time were recorded during the measurement. The first was the duration of the interruption itself. This time value should be similar to the previous measurement. The second time was the total outage time before the connection between the optical line termination (OLT) and the optical network unit (ONU) was re-established. A total of 30 measurements were performed, which gave huge differences between the individual results, with the maximum difference between the measured values being up to 37 s. The minimum value was 10 s, and the maximum was up to 47 s (see Figure 5). The outage time is determined by the size of the network, and it is a random process depending on the total number of end units with which the ONU establishes a connection.

### 3.3. Risk Analysis of Splitter/Coupler Insertion

There are two possible scenarios in this case. The inserted splitter/coupler is placed into an active optical network, where all traffic is encrypted and probably strictly monitored. This is the best possible scenario. On the other hand, it is possible to insert the splitter into the passive optical network in the upstream direction, immediately behind the already existing splitter. If it does not exceed the threshold for maximal attenuation, the detection of the attack will be more complicated. This is the worst possible scenario. The first scenario has a score of 9 points (2-1-3-3). This is a negligible risk considering traffic encryption, and the likelihood of data abuse is low. The second scenario has a score of 15 points (3-5-3-4). This risk is significant, and it is possible to abuse traffic data due to missing encryption (in a basic configuration).

### 3.4. Risk Minimization

Based on measurements, the most suitable splitter/coupler for the attack is with a splitting ratio above 90:10 due to the low inserted loss into the fiber path (see Table 4, Table 5 and Table 6). All attenuation changes higher than 0.3 dB should be violated as a part of the network monitoring strategy. From a theoretical point of view, attenuation changes may not occur. However, in real networks, they might occur, e.g., when the position of a fiber in a cassette is changed due to the installation of a new fiber, but the attenuation values differ maximally in a tenth of a decibel. It is also necessary to check the link status using a Simple Network Management Protocol (SNMP) or other network management methods and notify all link flaps, particularly for outages longer than five seconds. It is important to be able to highlight the interconnectedness of the mentioned phenomenon, which is a short unexpected network outage without any reason, followed by an insignificant increase in attenuation in the path. The system is responsible for the detection of such scenarios, or at least notifying an administrator with relevant information, which can help to identify a possible attack. In gigabit passive optical networks (GPON), it is favorable to check the received power of each customer unit and suspicious outages. The duration of the suspicious outage is evaluated and measured based on experiments.

### 3.5. Conclusions

The measurement of attenuation changes caused by splitter/coupler insertion shows that the lowest attenuation value during attack realization is approximately 0.3 dB. However, this value is dependent on the component quality and cleanliness. It is of interest to use connectors that are as clean as possible in order to reduce the insertion loss. Significant deviations occurred in the case of the 99:1 splitter measurement by the OTDR technique. From the measurement of time needed for splitter insertion, it is clear that the difference between point-to-point and point-to-multipoint networks is significant. Splitter insertion takes less than seven seconds. Moreover, insertion of a splitter with high-quality connectors takes less than 4 s. In the case of passive optical networks, particularly GPON, it is necessary to keep in mind the reconfiguration process, which means time window negotiation, key exchange, etc. This delay ranges from 6 to 41 s but mostly from 10 to 25 s. Insertion of the splitter/coupler proved to be a very effective attack. Of all the options verified, for some scenarios, it requires almost no special forms of preparation for its implementation, and insertion loss is very low when properly done. It is important to address the detection of these types of attacks and to implement protection and monitoring systems.

## 4. Leakage of Light Rays by Macrobending

**Figure 6:**
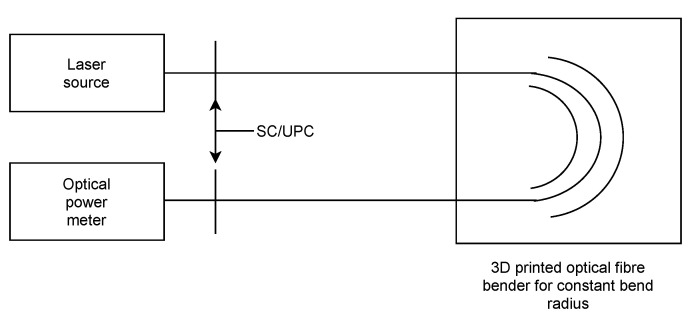
Scheme of attenuation measurement using the reference method.

The goal of this experiment is to evaluate the influence of the macrobend on fiber attenuation. The measurement scheme is shown in Figure 6. For the experiment, a G.652D optical fiber (standard telecommunication fiber) and two different schemes for results verification were used. The first experiment used the reference measurement method, and the second experiment used GPON OLT and the internal statistics of each connected device. Macrobending was achieved using plates with grooves with various radii printed by a 3D printer. The fiber only with primary protection was inserted between plates.

Both experiments used the same equipment: a Huawei Technologies Co. Ltd., GPON-OLT Class C+ SFP module, Made in Shenzhen, China connected to the TP-Link Technologies Co., Ltd., TP-Link MC200L optical converter, Made in Shenzhen, China and Shenzhen NOYAFA Electronic Co., Noyofa DXP-40D optical power meter, Made in Shenzhen, China. Both devices had SC connectors. The accuracy of this measurement was 0.01 dB because of the power meter limitation.

**Figure 7:**
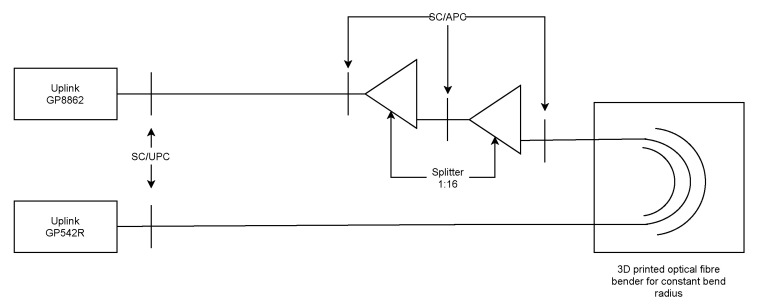
Scheme of attenuation measurement using the OLT unit.

Measurement by GPON OLT used a fully functional GPON network with the same SFP module as in the previous experiment in order to adequately compare the results. The GPON network consisted of an Uplink GP8861 OLT and Uplink GP542R ONUs. The OLT provided attenuation values with an accuracy of 0.001 dBm for each connected ONU; thus, it was possible to evaluate the attenuation caused by the macrobend (see Figure 7).

This setup required two 1:16 splitters connected consecutively to provide enough attenuation for successful communication of the OLT and ONU. The measurement results are shown in Table 7.

### 4.1. Risk Analysis of Macrobending

Evaluation of the attack obligation is strongly dependent on the fiber type and network type. Assuming that G.652D fibers are used mostly in WAN/MAN PTP networks due to minimal dispersion at a 1310 nm wavelength, the traffic is encrypted and the network contains strong monitoring. The advantage of such an attack is that it is place-independent. Based on experience, this attack causes more harm than good because the fiber needs to be stripped of all layers of protection without breaking connections or causing an outage. Another possible problem is the necessity of a very sensitive photodetector to sample the light emission that escapes from the fiber. This type of attack is more theoretical or laboratory-based. In each evaluated category, the attack has a score of 10 (5-1-5-1), but the amount of captured data is limited by the bend attenuation.

### 4.2. Risk Minimization

The optical fiber should be installed as straight as possible with a constant bend with a high radius to minimize additional attenuation. The fiber should also be protected against any external influences, including attackers. Considering that the attack may be performed anywhere on the fiber path, it is necessary to minimize the access possibilities for the attacker to any segment of the infrastructure. It is also wise to encrypt all network traffic and monitor any higher and unexpected attenuation changes over 3 dB. The attacker may try to penetrate the infrastructure without success. In such a case, it is necessary to verify this location and secure the path. In places where the fiber is more accessible (FTTx infrastructures provide fiber ends directly to customers, which is more accessible than other topologies), it is recommended to use a fiber that is resistant to bending (e.g., G.657).

### 4.3. Conclusions

This section presents the results of fiber macrobending measurement and attenuation for various bend radii of the fiber. According to these results, the critical threshold for the G.652D fiber is 14 mm, after which the attenuation rapidly increases. It can be assumed that the threshold has exponential growth until the fiber is broken, which is at a radius of 7 mm.

## 5. Light Back-Reflection

**Figure 8:**
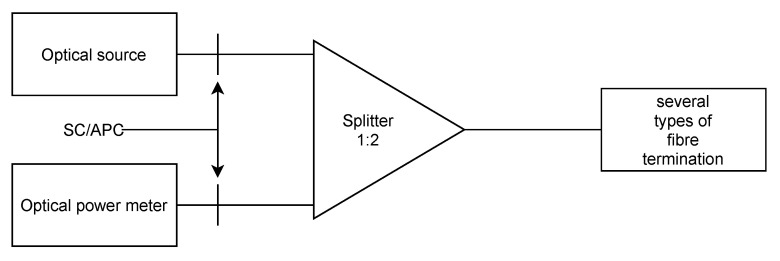
Scheme for back-reflection measurement.

This measurement investigated the influence of the used connectors on back-reflection in GPON networks (the scheme is depicted in Figure 8). This eavesdropping method might be used in the GPON networks to capture unencrypted traffic in the downstream direction. Advanced Encryption Standard (AES) encryption is optional in the GPON network and is often not used, which may lead to attempts at such attacks.

For the measurement, a TP-Link MC220L media converter with a Huawei GPON SFP module, Noyafa DXP-40D optical power meter, 1:2 optical splitter, and SC/APC and SC/UPC adapters were used. Results are given in Table 8. The optical signal in the downstream direction used a 1490 nm wavelength. ONUs transmitted a signal at a 1310 nm wavelength; therefore, for the back-reflection measurement, the signal had to be filtered out.

### 5.1. Risk Analysis of Back-Reflection

This type of attack is defined for passive optical networks, where the upstream traffic is unencrypted (but can also be applied to point-to-point networks). The attack requires filtering out the downstream signal from the upstream reflected signal and requires amplification of the signal. It is very likely that the attack remains unrevealed. The attack has a score of 16 points (5-5-1-5) in an unencrypted GPON network and a score of 12 points (5-1-1-5) in a GPON network with encryption enabled. The risk is significant and may be simply minimized.

### 5.2. Risk Minimization

The attack is difficult in terms of hardware because the attacker needs to separate the reflected upstream signal from the downstream signal and amplify it. However, the attack is plausible and it is appropriate to minimize the risk. Data also do not come only from a single ONU, which makes the eavesdropping process more difficult for a single device. The most suitable risk minimization method is to enable encryption with a symmetrical cipher (e.g., AES). The cipher is optional in the GPON network, which is fixed in newer versions of xPON recommendations. The next possible mitigation is to avoid attacks and access to the infrastructure or the network termination of each path. These are often unused connections located in a house basement. This problem may be solved by unplugging unused fibers from the splitter. Unfortunately, it is not possible to avoid the scenario in which an attacker is one of the connected customers, but this scenario may be covered by enabling traffic encryption.

### 5.3. Conclusions

The back-reflection method focuses on the measurement of the back-reflection of various types of commonly used connectors, particularly SC. From the results, there is a clear difference between narrow ultraphysical contact (UPC) and angled physical contact (APC) ferrules. The differences are 16.65 dB for unplugged connectors and 14.14 dB for plugged connectors. The difference between plugged-in and unplugged connectors is obvious but it is likely that the scenario with unplugged connectors will not be as frequent.

## 6. Crosstalk in Wavelength Multiplexers

Crosstalk is an undesirable phenomenon arising on both active and passive elements of optical transmission systems. The measurement was focused on spectral analysis and evaluation of crosstalk. The wavelength multiplexing increases the transmission bandwidth by transmitting multiple wavelengths in a single optical fiber. Passive components frequently used for wavelength multiplexing may cause crosstalk between individual outputs/channels because of their limited isolation.

### 6.1. Spectral Analysis of Crosstalk in a DWDM Arrayed Waveguide Grating Multiplexer

**Figure 9:**
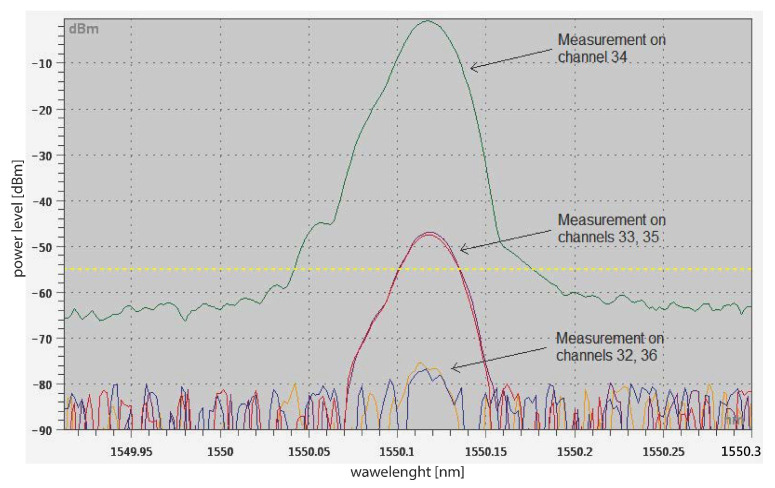
Channel crosstalk of AWG DWDM MUX.

The goal was to determine how much crosstalk occurs on neighboring channels in dense wavelength division multiplexing (DWDM) networks. An integrable tunable laser (ITLA) with a precisely adjustable wavelength and output power, a sixteen-channel DWDM Arrayed waveguide grating (AWG) MUX (channels 26–41 according to International Telecommunication Union), and an optical spectrum analyzer OSA20 from Yenista Optics with a resolution of 20 pm were used for the measurement. The input signal wavelength was set to 1550.12 nm, corresponding to channel 34. Subsequently, measurements were performed on the side channels (spacing 100 GHz, i.e., channels 33 and 35) and channels shifted by 200 GHz (channels 32 and 36). Measured crosstalk can be seen in Figure 9.

For adjacent channels, crosstalk was approximately down to 47 dB. Crosstalk from the channel at a grid of 200 GHz was measurable but the power level was low so the possibility for the subsequent processing of the signal was minimal.

### 6.2. Dependency of Input Signal Power Level on Crosstalk in DWDM AWG MUX

**Figure 10:**
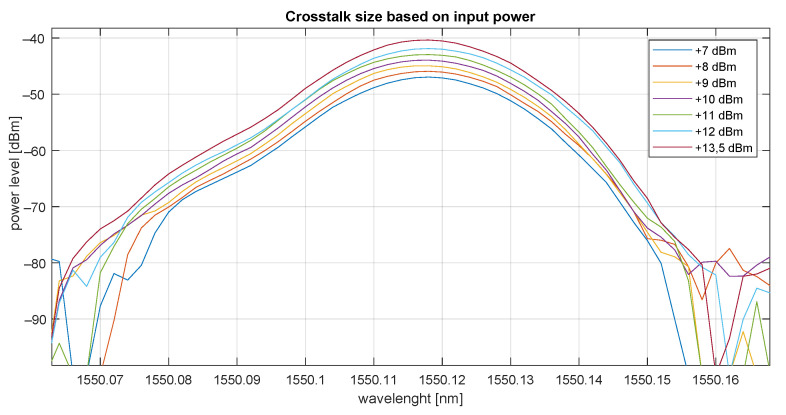
Input power level vs. crosstalk.

The dependency of the signal intensity on crosstalk was verified using a laser with variable output power ranging from +6 to +13.5 dBm. Crosstalk was measured between channels 34 and 33 (channel 34 corresponds to 1550.12 nm). Based on the measurement (see Figure 10), it is obvious that there is a linear dependency and crosstalk is approx 40 dB. Crosstalk for different laser power levels is shown in Table 9.

### 6.3. Spectral Analysis of 1×2 CWDM ADD/DROP

**Figure 11:**
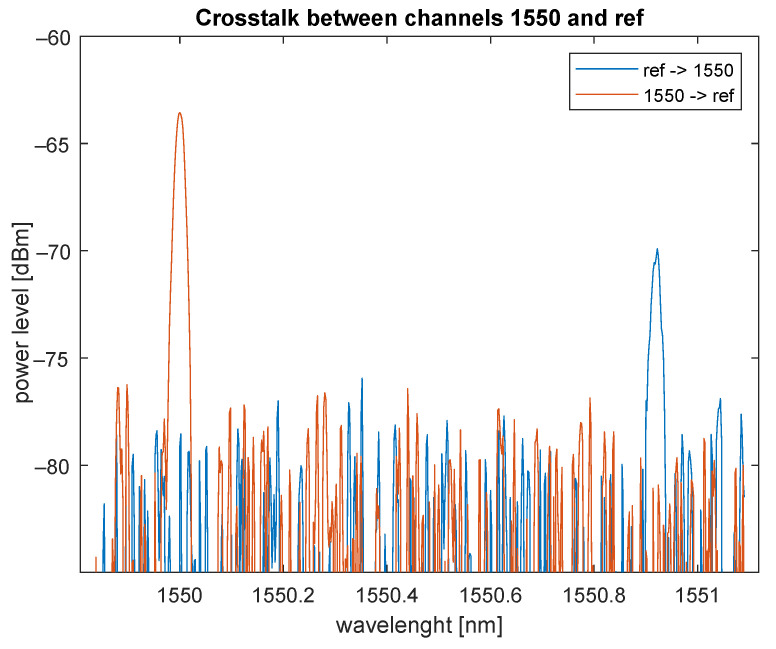
Crosstalk between REF and PASS ports in CWDM ADD/DROP.

By the measurement complex analysis of standard coarse wavelength division multiplexing (CWDM), ADD/DROP MUX for a 1550 nm grid was performed. The output power was set to +6 dBm. Different wavelengths in a 20 nm CWDM channel grid were used only for better visualization, i.e., the possibility of plotting both signals in one graph. The graph in Figure 11 shows the measured crosstalk between the REF (reflected) port and PASS (1550) port, and one can see that crosstalk was close to the noise level. If the attacker tries to filter out the wavelength outside the CWDM range from the ADD/DROP MUX, it can be assumed that it would be almost impossible to obtain useful information.

**Figure 12:**
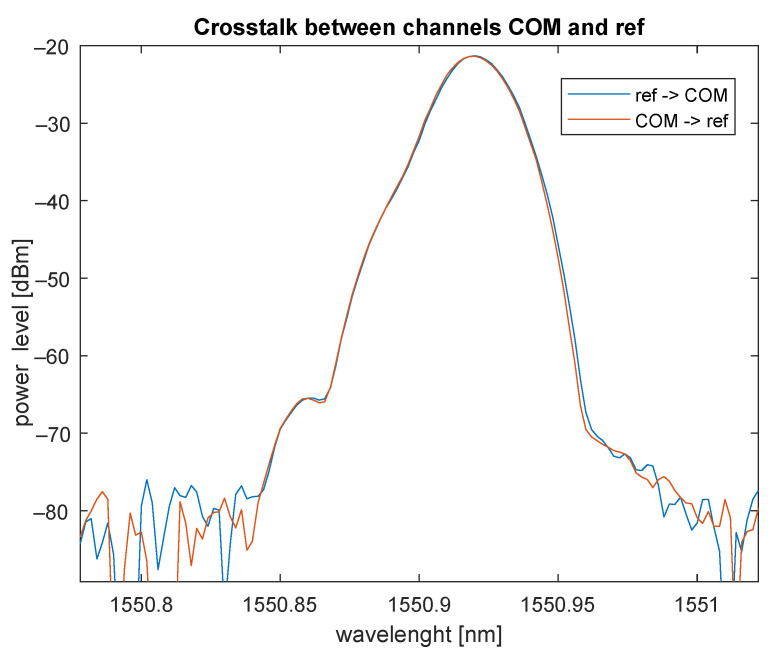
Crosstalk (islolation) between REF and COM ports in CWDM ADD/DROP.

Figure 12 shows the standard functionality of filtering out (drop) the required wavelength (1550 ± 20 nm in our case). Isolation between COM and REF ports and vice versa is approximately 27 dB.

### 6.4. Risk Analysis of ADD/DROP or MUX

For ADD/DROP or MUX crosstalk attacks, it is very likely that all traffic is encrypted considering the usage of this component. ADD/DROP or MUX are used to increase the bandwidth and capacity of already existing optical networks. For the attack, it is not necessary to add any additional device to the network or interrupt the path. On the other hand, the crosstalk signal is, in some cases, very weak, and, without any amplification (due to the magnitude of the suppression, the use of an amplifier is also limited), it is not possible to process the signal. ADD/DROP or MUX crosstalk achieves 12 points (5-1-1-5) on the risk scale, which is not a negligible risk and should be handled properly.

### 6.5. Risk Minimization

The measurement proves that crosstalk in wavelength multiplexing components can be used for eavesdropping. To avoid crosstalk eavesdropping, lower output power and high-quality components with high isolation should be used.

### 6.6. Conclusions

The measurement focused on crosstalk in wavelength multiplexing components proves the existence of such risk in DWDM and in CWDM networks. The crosstalk in DWDM in two neighboring channels is approximately 47 dB, and, in the case of crosstalk over two neighboring channels, it is approximately 77 dB. Furthermore, the measurement proves that the relation between the signal power level and crosstalk level is linear and directly proportional. Crosstalk in CWDM components is approximately 65 dB and isolation is therefore sufficient to minimize the possibility of attack.

## 7. Crosstalk in Optomechanical Switch

**Figure 13:**
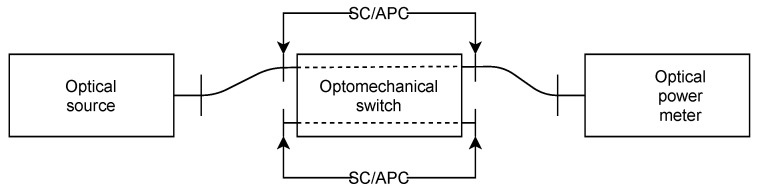
Measurement scheme for an optomechanical switch.

An optomechanical switch is an active device capable of switching light emissions from input ports to selected output ports. The goal of this measurement (measurement scheme is depicted in Figure 13) was to verify the parameters of such a component, mainly crosstalk between output ports. The measurement setup uses a media converter TP-Link MC200L with a 1550 nm Ubiquiti SFP module, a Noyafa DXP-40S optical power meter with a sensitivity range from +10 to –70 dBm, and an optomechanical optical switch 2 × 2 connected to the breadboard using 10-cm-long DuPont jumpers to choose its switching settings.

The measurement shows that the measured IL differs by a maximum of 0.3 dB compared to the datasheet. A higher value than the datasheet may be due to a measurement error or because of connectors; however, in some cases, the value is lower than the value specified in the datasheet; see Table 10. Regarding eavesdropping, the datasheet claims that the isolation is higher than 55 dB (similar switches from the competition have isolation higher than 70 dB). The measurement shows no crosstalk in any port combination. Considering the power meter sensitivity, which is −70 dBm, there is undetected crosstalk. Therefore, assuming that optomechanical switches are secured devices (in terms of crosstalk), the largest weakness is the possibility of local/remote changes in the output port. The entirety of the traffic can be redirected, but this is simply detected because of a denial of service.

### 7.1. Risk Analysis of Optomechanical Switch

Considering the high port isolation, i.e., low crosstalk, this type of information attack is very unlikely. It is likely destructive and achieves a score of 8 (1-1-5-1).

### 7.2. Risk Minimization of Optomechanical Switch

Based on the results, attacks on this device are not focused on eavesdropping but on the denial of service. Access to critical infrastructure components, such as optomechanical switches, must be minimized, not only physically but also remotely.

### 7.3. Conclusions

Optomechanical switches are notable for their high port isolation and very low insertion loss, which was also experimentally proven. IL was less than 1 dB and crosstalk was more than 70 dB. The only possible weakness is the swapping of switch pins, which can also be done remotely.

## 8. Conclusions

The paper deals with the issue of information leakage through passive and active elements of optical fiber infrastructure. Measurements of the most common attack scenarios, including the insertion of an optical splitter/coupler, macrobend loss measurement, eavesdropping based on crosstalk in multiplexing components and an optomechanical switch, and also eavesdropping based on the back-reflection, were evaluated. It is clear from the measurements that most attacks are feasible; however, for example, the isolation between channels in CWDM ADD/DROP is too high and the obtained signal is too weak for further amplification/processing. Likewise, the isolation between ports of the optomechanical switch is sufficient to ensure secure data transmission. The largest potential danger is inserting a 1:99 power splitter into the route. The time required to carry out an attack is relatively small and the insertion loss is almost undetectable.

## Figures and Tables

**Table 1:** Insertion loss of the coupler 80:20.

Port IN	Port OUT	IL from Datasheet (dB)	IL at 1310 nm (dB)	IL at 1550 nm (dB)
P1	P4	7.13	7.21	7.11
P1	P3	1.44	1.56	1.43
P2	P3	1.28	1.63	1.58
P2	P3	7.06	7.79	7.38

**Table 2:** Insertion loss of the coupler 90:10.

Port IN	Port OUT	IL from Datasheet (dB)	IL at 1310 nm (dB)	IL at 1550 nm (dB)
P1	P4	10.58	10.58	10.20
P1	P3	0.68	0.68	0.65
P2	P4	0.72	0.79	0.79
P2	P3	10.36	10.45	9.93

**Table 3:** Insertion loss of the coupler 99:1.

Port IN	Port OUT	IL from Datasheet (dB)	IL at 1310 nm (dB)	IL at 1550 nm (dB)
P1	P4	20.47	22.50	20.66
P1	P3	0.37	0.35	0.23
P2	P4	0.33	0.31	0.26
P2	P3	20.43	21.91	20.14

**Table 4:** Insertion loss of the coupler 80:20 measured with OTDR method.

Port IN	Port OUT	IL from Datasheet (dB)	IL at 1310 nm (dB)	IL at 1550 nm (dB)
P1	P3	7.13	7.28	6.15
P1	P4	1.44	1.72	1.70
P2	P3	1.28	1.63	1.56
P2	P4	7.06	7.09	5.77

**Table 5:** Insertion loss of the coupler 90:10 measured with OTDR method.

Port IN	Port OUT	IL from Datasheet (dB)	IL at 1310 nm (dB)	IL at 1550 nm (dB)
P1	P3	10.58	10.42	9.85
P1	P4	0.68	0.81	0.77
P2	P3	0.72	0.71	0.64
P2	P4	10.36	10.86	10.09

**Table 6:** Insertion loss of the coupler 99:1 measured with OTDR method.

Port IN	Port OUT	IL from Datasheet (dB)	IL at 1310 nm (dB)	IL at 1550 nm (dB)
P1	P3	20.47	18.35	15.90
P1	P4	0.37	0.37	0.28
P2	P3	0.33	0.39	0.36
P2	P4	20.43	18.31	15.35

**Table 7:** Measured values of bending radius.

	Noyafa DXP-40D	Uplink GP8862 + GP542R
Bending Radius [mm]	IL [dB]	IL [dB]
20	0.07	0.178
19	0.16	0.248
18	0.23	0.332
17	0.42	0.558
16	0.56	0.902
15	0.71	1.216
14	0.99	1.396
13	1.39	2.866
12	3.71	3.218
11	6.35	4.918
10	8.76	8.306
9	11.55	10.15
8	15.78	17.007

**Table 8:** Measurements of backscattering.

Type of Fiber Termination	Reflection [dB]
Connected SC/APC	68.03
Unconnected SC/APC	56.91
Connected SC/UPC	51.38
Unconnected SC/UPC	45.77
Without connector	31.61

**Table 9:** Crosstalk for different laser power levels.

Output Power [dBm]	Crosstalk [dB]
7	54.096
8	54.056
9	54.104
10	54.105
11	54.067
12	53.984
13.5	54.019

**Table 10:** Insertion loss of optomechanical switch.

1550 nm	IL from Datasheet [dB]	Measured IL [dB]
P1–P3	0.96	0.65
P1–P4	1.01	0.82
P2–P4	0.59	0.89
P2–P3	0.71	0.99
